# Corrigendum: Quantum State Transmission in a Superconducting Charge Qubit-Atom Hybrid

**DOI:** 10.1038/srep46910

**Published:** 2017-12-22

**Authors:** Deshui Yu, María Martínez Valado, Christoph Hufnagel, Leong Chuan Kwek, Luigi Amico, Rainer Dumke

Scientific Reports 6: Article number: 38356; 10.1038/srep38356 published online: 12 06 2016; updated: 12 22 2017.

This Article contains errors in the legend of Figure 3.

ψ_c_ = μ|a〉 + ν|b〉

should read:

ϕ_c_ = μ|0〉 + ν|1〉

In addition,

ϕ_a_ = μ|a〉 + ν|b〉

should read:

ψ_a_ = μ|a〉 + ν|b〉

